# Predictors of fear control related to COVID-19 among older population: an investigation on COVID-19 risk perception and health related quality of life during the pandemic

**DOI:** 10.1186/s12955-023-02167-1

**Published:** 2023-07-29

**Authors:** Saeedeh Avazzadeh, Neda Gilani, Leila Jahangiry

**Affiliations:** 1 Medical Education Research Center, Health Management and Safety Promotion Research Institute, Tabriz University of Medical Sciences, Tabriz, Iran, Tabriz University of Medical Sciences, Azadi Street, Golgasht Street, Tabriz, Iran; 2 Department of Statistics and Epidemiology, Faculty of Health, Tabriz University of Medical Sciences, Tabriz, Iran; 3 Health Education and Health Promotion Department, Faculty of Health, Tabriz University of Medical Sciences, Tabriz, Iran

**Keywords:** Covid-19, Health related quality of life, Risk perception, Older adults, Danger control, Fear control

## Abstract

**Background:**

The aim of this study was to examine the role of demographic characteristics, general health and health related quality of life on the fear control. Also, the aim of study was to explore how older people percept the COVID-19 pandemic by using the component of the expanded parallel process model (EPPM), and how the possible perception may contribute to probable behavior responses to prevention and control of COVID-19.

**Methods:**

The present study is a cross-sectional study that was conducted in Tabriz, a city in north of Iran from February to April 2021, the period that correspond with the fourth wave of COVID-19. To collect information, the Covid-19 risk perception questionnaire (based on EPPM model including efficacy, defensive responses, and perceived threat) and health related quality of life (HQOL) Short Form-36 questionnaire were used. Discriminate value was calculated to estimate fear control and danger control. Univariate and multivariable logistic regression were calculated to examine the effect of demographic characteristics, general health and health-related quality of life on the fear control.

**Results:**

The mean age of the 350 participants was 67.9 (6.4) years. A total of 83.1% of participants were engaged in danger control processes and 16.9% in fear control processes. According to the multivariable results, significant predictors for fear control were: gender 1.57 (95% CI 1.05–2.34, 0.025), education 7.38 (1.42–38.35, *p* = 0.017), economic status 1.31 (0.4–0.63, *p* = 0.029), and significant protective factors for fear control were: body pain 0.97 (0.94–0.99, *p* = 0.041), general health 0.96 (0.93–0.98, *p* = 0.032), physical health 0.94 (0.90–0.98) and total quality of life 0.024 (0.89–0.98).

**Conclusions:**

Strong associations of fear control were found with being female, being illiterate, and having a good economic status and also body pain, general health, physical health and total HQOL were significantly associated with danger control. Since, most of elderly populations have adequately higher perceptions of efficacy to counteract their threat perceptions to continue motivating these older people to engage in COVID-19 self-protective behaviors, it is necessary to emphasis on the susceptibility of target population and the severity of the COVID-19 threats.

## Background

The coronavirus pandemic (COVID-19) has initiated as a major global health challenge. Efforts to identify the first case of a COVID-19 infection was reported in Wuhan, China, in December 2019 [1]. The COVID-19 disease has had a huge impact on individuals all around the world especially on older individuals. Such significant changes are necessarily followed by anxieties caused from variety aspects of life [2]. The most notable of which are health and financial instability. The COVID-19 raises the risk of mortality and significant problems as people become older. The rise in death rates in persons with pre-existing medical problems (severe chronic diseases, such as heart disease) was an additional factor burdening the senior population [3].

The ongoing global pandemic of COVID-19 has been further complicated by the emergence of new strains of the virus. These new strains, such as the Alpha, Beta, Gamma, and Delta variants, have been identified in various parts of the world, contributing to increased transmissibility and potential challenges in public health response efforts. These strains are believed to have different characteristics, including higher infectivity rates or potential resistance to certain treatments or vaccines. As a result, they have led to increased concern among health authorities and governments, necessitating the implementation of stricter measures to contain the spread of the virus [4, 5].

Elderly people make up on average 16 percent of the population in industrialized countries and this number is expected to rise to 19 percent in the next decades [6]. Iran is experiencing a rapid transition towards becoming an aging society, with one of the fastest growth rates of older population globally. According to available data, the proportion of elderly individuals in Iran surged from 7.22% in 2006 to 20.8% in 2011, indicating a significant increase in just five years. It is projected that this trend will continue, and by 2050, the elderly population is expected to reach 21.7% [7, 8]. This demographic shift poses various challenges for the country, including healthcare, pension systems, and social support. According to data from the Oxford COVID-19 Evidence Service, persons in their 60s have a mortality risk of 3.6 percent which rises to 8.0 and 14.8 percent in their 70s and 80s, respectively [9]. Approximately 80% of COVID-19-related deaths have occurred in adults over the age of 60. According to US reports, ten to twenty-seven percent of persons over the age of 85 are in the risk of death because of COVID-19 [10, 11].

Risk perception as a common psychological reaction is a subjective assessment of the likelihood of bad events made by an individual [12]. It is affected by cognitive, emotional, social, and cultural variables [13].

Risk perception related to COVID-19 among older individual are reflected in the insecurities and impact adversely on the quality of life of older persons who believe COVID-19 poses a significant risk to their health or financial status [14]. In general, aging might increase one's susceptibility to certain diseases and lead to impairment in later years [15, 16]. Given the increased vulnerability of older persons to the COVID-19 and detrimental impacts of aging on one's capacity, their dependence will raise the need for help during COVID-19 pandemic.

World Health Organization (WHO) announced that every country should develop and implement comprehensive public health measures to combat the spread of COVID-19 [17]. Available findings show that temporary mobility restrictions, social distance, and large-scale gathering ban, in combination of wearing mask and cleaning hands help reduce the spread of COVID-19, but they also have negative consequences in many aspects of individual’s life [18].

Older persons are experiencing a generational divide on how they encourage to separate themselves from other people and self- isolate to stop the spread of COVID-19 [19]. The likely increase in the incidence of serious health complications from covid-19 among older adults, especially those with comorbid conditions, has exacerbated their physical and social isolation, and led to increase in the prevalence and severity of anxiety and depression among this population group [20]. Social isolation increase the risk of depression in older people [21].

Strong evidences show that several factors may affect COVID-19 related behaviors [22–24]. According to health education theories, risk perception is one of key factors that influence health behaviors because an individual perceived threat to prevent disease [25]. Of these, the expanded parallel process model (EPPM) is a relevant theory that explains how fear messages persuade people into healthy behavior and determine that under which circumstances fear messages succeed or fail [26]. Many investigations have found a link between fear emotions and heightened risk perception with frightened people seeing a higher chance of bad outcomes after being exposed to fear-related stimuli. This link has been discovered in a variety of people, including those who are afraid of snakes and spiders [27–29], those who are afraid of contamination [30], and those who are socially nervous [31].

The aim of this study was to examine the role of demographic characteristics, general health and health related quality of life on the fear control. Also, the aim of study was to explore how older people percept the COVID-19 pandemic by using the component of the EPPM model (efficacy, defensive responses, perceived threat), and how the possible perception may contribute to probable behavior responses to prevention and control of COVID-19.

## Methods

### Study design and participants

The present study is a cross-sectional study that was conducted in Tabriz, a city in north of Iran from February to April 2021, the period that correspond with the fourth wave of COVID-19 in Iran. The study recruited the elderly individuals over 60 years of age [32]. The inclusion criteria were: being mentally conscious and able to answer the study questions. Cluster sampling method was applied for sampling. Tabriz has sixteen health centers that considered as the study clusters. The health records of older people were available in the health centers. Four health centers were randomly selected from sixteen centers and then about 25% of the blocks were selected from the selected centers. In the next stage, the phone number of older individuals were extracted from their health records and through phone interview they were asked to participate in the study. Then, eligible and interested participants were invited to the study through a telephone call interview by a trained researcher (SA, MSc student in healthy aging).

### Measurements and scoring

To collect information, the Covid-19 risk perception questionnaire [23], and HQOL Short Form-36 questionnaire were used. The Covid-19 risk perception questionnaire is a 29 items questionnaire contemplating three predefined dimensions including: self-efficacy, response efficacy, defensive responses (avoidance, denial, and reactance), and threat perception (susceptibility and severity). The perceived self-efficacy consisted of six items measuring participants’ beliefs about their ability to perform the recommended responses to the COVID-19. Response efficacy included seven items to assess participants’ opinion about the effectiveness of the recommended preventative responses to the COVID-19. Defensive response (denial, reactance and avoidance) included eight items to investigate people’s beliefs about their perception of the risk of COVID-19. Threat perception included eight items and measured people’s beliefs about the extent of the COVID-19 risk and about their risk of experiencing the disease. A 5-point Likert scale (1 = strongly disagree, 2 = disagree, 3 = neutral, 4 = agree, 5 = strongly agree) was used to rate the scores and an overall row score ranging from 29 to 145. The row scores were converted into a score of 0 to 100 where lower scores indicated lower risk perception and the higher scores indicated higher risk perception. The risk percept questionnaire is well-designed among Iranian population [23]. Danger control and fear control were estimated by subtracting the perceived threat score from the perceived efficacy score (self- plus response-efficacy divided by two), resulting in a discriminating value. In the current estimation, a positive value presents that a person was engaging in danger control because she/he have had higher perceived efficacy score than perceived threat score. A negative value shows that a person was engaged in fear control because of having higher threat perception score than perception of efficacy.

The SF-36 QOL questionnaire is a generic measurement to assess self-reported health status, functioning and well-being. The psychometric properties of the Iranian version of SF-36 are well documented [33].

Demographic information were questions about (age, gender, history of COVID-19, having hypertension, having diabetes, having cardiovascular disease (CAD).

### Statistical analysis

The normality of the data was assessed using a Kolmogorov–Smirnov test and the normal distribution of data was confirmed. The characteristics of the participants were summarized as numbers, percentages, or means with standard deviations, where appropriate. Analysis of variance (ANOVA), chi-square and t-tests were used to compare the study sub-groups. In all tests, a value of *p* < 0.05 was considered statistically significant. Descriptive analysis was done by tabulation of data and presentation of continuous variables as means and standard deviations and categorical variables as numbers and proportions. Various effect measures are available for quantifying the relationship between an intervention or a risk factor and an outcome, such as the risk ratio and the odds ratio. Odds ratios are intended for use in case–control and cross-sectional studies. Univariate and multivariable logistic regression were calculated to examine the effect of demographic characteristics, general health, and health-related quality of life on the fear control. In order to evaluate the multivariable logistic regressions, we used Receiver Operating Characteristic (ROC) curves and correctly classified values. The closer these values are to one hundred (incorrectly classified value) and one (in ROC curve), the better the model fits. Data analysis was done using STATA v17 (College Station, TX).

## Results

### Demographic characteristics

Table 1 presents the descriptive characteristics of the participants. The mean age of the 350 participants was 67.9 (6.4) years. Men accounted for (59.4%) of study population. In the sample, 86% of the participants were married (86%) and (30.5%) were illiterate. In total 17.7% of the participants reported that they had high blood pressure, 10.3% and 9.4 suffered from diabetes and cardiovascular disease, respectively. In this study, 15.2% stated having two or more comorbidities and 17.7% reported the history of getting COVID-19.

**Table 1:** Demographic characteristics of participants

	Total N (%)	60–70 N (%)	71 ≥ N (%)
**Gender**			
Male	208 (59.4%)	147 (58.8%)	61 (61%)
Female	142 (40.6%)	103 (41.2%)	39 (39%)
**Marital status**			
Single	2 (.6%)	2 (.8%)	0 (0%)
Married	301 (86%)	224 (89.6%)	77 (77%)
Widowed/divorced	47 (13.4%)	24 (9.6%)	23 (23%)
**Educational status**			
Illiterate	106 (30.5%)	75 (30.4%)	31 (31%)
Elementary education	83 (23.9%)	55 (22.3%)	28 (28%)
School degree	59 (17%)	41 (16.6%)	18 (18%)
Diploma	61 (17.6%)	45 (18.2%)	16 (16%)
University	38 (11%)	31 (12.6%)	7 (7%)
**Economic status**			
Poor	145 (41.5%)	102 (40.8%)	43 (43%)
Intermediate	151 (43.1%)	109 (43.6%)	42 (42%)
Good	54 (15.4%)	39 (15.6%)	15 (15%)
**Having chronic disease**			
Hypertension (yes)	62 (17.7%)	40 (16%)	22 (22%)
Diabetes (yes)	36 (10.3%)	29 (11.6%)	7 (7%)
CAD	33 (9.4%)	23 (9.2%)	10 (10%)
Comorbidity	53 (15.2%)	37 (14.8%)	16 (16%)
History of getting COVID-19	49 (14%)	37 (14.8%)	12 (12%)
History of getting COVID-19 in family members	62 (17.7%)	44 (17.6%)	18 (18%)

*CAD* Cardiovascular disease

### Risk perception and health related quality of life status

Results of risk perception are shown in Table 2. The statistically significant differences were observed between age categories for efficacy, defensive responses (denial, avoidance, and resistance), and susceptibility.

**Table 2:** Risk perception scores related to COVID-19 among elderly population

Variable	60–70 years	71 years ≥	*P*-value
	Mean (SD)	Mean (SD)	
**Response efficacy**			
Efficacy	98.3 (6.2)	97.3 (8.7)	.015
Self-efficacy	97.2 (7.8)	96.3 (10.2)	.122
**Defensive responses**			
Denial	98.1 (7.2)	95 (13.3)	.0001
Avoidance	96.3 (9.5)	94.7 (12.4)	.014
Resistance	97.3 (8.8)	95.8(10.5)	.011
**Threat**			.775
Susceptibility	97.3 (8.2)	96 (10.6)	.015
Severity	97.5 (10.3)	97.1 (8.7)	.371

The comparison of health related quality of life among age groups (60–70 and 71 ≥) are presented in Table 3. Physical function is significantly different between the two groups (*p* = 0.0001), and more in the age group of 60–70 years. The Role-physical is significantly different between the two groups (*p* = /001), and more in the age group of 71 years and older. There is no significant difference between the other variables in the two age groups.

**Table 3:** Health related quality of life of elderly participants during COVID-19 pandemic

Variable	60–70	71 ≥	*P*-value
	Mean (SD)	Mean (SD)	
Physical Function	87.5 (21.7)	77.0 (34.1)	.0001
Role-Physical	16.2 (35.1)	23.7 (41.3)	.001
Body Pain	80.5(22.1)	78.4 (24.0)	.242
General Health	56.9 (12.8)	56.3 (14.4)	.331
Vitality	65.4 (16.5)	68 (17.6)	.342
Social Functioning	77.5 (20.6)	76.3 (24.2)	.032
Role Emotional	24.25 (41.8)	21.6 (40.6)	.497
Mental Health	67.6 (14.3)	71.2 (14.4)	.803
Physical Health	61.1 (8.2)	60.4 (10.1)	.029

The discriminating values to indicate danger control and fear control based on socio-demographic characteristics of participants were estimated (Table 3). A total of 83.1% of participants were engaged in danger control processes and 16.9% in fear control processes. The elderly participants in danger control group were more likely to deal with efficacy of recommended responses for prevention of COVID-19.

Table 4 presents the results of univariate and multivariable modeling, including odds ratios and their corresponding 95% confidence interval for the association between the demographic characteristics, general health, and health-related quality of life with the fear control. According to the multivariable results, gender, education, economic status, body pain, general health, physical health and total quality of life were significant predictors of fear control. The women were 1.57 times more likely to have fear control (*P* = 0.025). Participants with illiterate education were 7.38 times more likely than other ducation level to show fear control (*P* = 0.017). Also, People with good economic status were 2.70 times more likely to the fear control than intermediate status (*P* = 0.029). Increasing of body pain (P = 0.041), general health (0.032), physical health (*P* = 0.026), and total quality of life (*P* = 0.024) had protective effect on the fear of control.

**Table 4:** Association the demographic characteristics, general health and health related quality of life with the fear of control

	Danger control N (%)	Fear control N (%)	Odds Ratio (95% CI)	P	Adjusted Odds Ratio^a^ (95% CI)	P
	291 (83.1)	59 (16.9)				
**Sex**						0.025
Male	180 (61.9)	28 (47.5)	Ref		Ref	
Female	111 (38.1)	31 (52.5)	1.80 (1.02,3.15)	0.042	1.57 (1.05,2.34)	
**Age (year)**						0.761
60–70	208 (71.5)	42 (71.2)	Ref		Ref	
> = 71	83 (28.5)	17 (28.8)	1.01 (0.55,1.88)	0.984	1.12 (0.55,2.57)	
**Marital status**						0.938
Married			Ref		Ref	
Single/Widowed/ divorced			1.32 (0.62,2.82)	0.475	1.04 (0.40,2.71)	
**Education**						
Illiterate	78 (27.1)	28 (47.5)	6.46 (1.46,28.61)	0.014	7.38 (1.42,38.35)	0.017
Elementary	65 (22.6)	18 (30.5)	4.98 (1.09,22.71)	0.038	4.50 (0.90,22.65)	0.068
School degree	52 (18.1)	7 (11.9)	2.42 (0.48,12.34)	0.287	2.14 (0.38, 11.93)	0.386
Diploma	57 (19.8)	4 (6.8)	1.26 (0.21,7.25)	0.793	1.67 (0.27,10.43)	0.580
University	36 (12.5)	2 (3.4)	Ref		Ref	
**Economic status**						
Poor	119 (39.9)	26 (44.1)	1.52 (0.80,2.88)	0.202	1.31 (0.47, 0.63)	0.469
Good	40 (13.7)	14 (23.7)	2.43 (1.12,5.28)		0.025	0.029
Intermediate	132 (45.4)	19 (32.2)	Ref		Ref	
**Hypertension**						0.224
Yes	92 (31.8)	23 (39)	1.37 (0.77, 2.44)	0.289	1.57 (0.75, 3.23)	
No	197 (68.2)	36 (61.0)	Ref		Ref	
**Diabetes**						
Yes	74 (25.6)	15 (25.4)	0.99 (0.52, 1.88)	0.977	0.71 (0.31,1.58)	0.394
No	215 (74.4)	44 (74.6)	Ref		Ref	
**CAD**						
Yes	46 (15.9)	9 (15.3)	0.95 (0.44, 2.07_	0.899	1.10 (0.42, 2.83)	0.850
No	243 (84.1)	50 (84.7)	Ref		Ref	
**History of getting COVID-19**						
Yes	35 (12)	12 (20.3)	0.53 (0.26, 1.11)	0.092	1.52 (0.59.3.89)	0.382
No	256 (88)	47 (79.7)	Ref		Ref	
**History of COVID-19 in family members**						0.305
Yes	47 (16.2)	15 (25.4)	0.57 (0.29, 1.10)	0.092	1.59 (0.65, 3.88)	
No	244 (83.8)	44 (74.6)	Ref		Ref	
	Mean (SD)	Mean (SD)				
Physical Function	84. (26.7)	86.6 (23.9)	1.00 (0.98,1.02)	0.493	1.01 (0.99,1.03)	0.233
Role-Physical	19.1 (37.4)	14.4 (35.1)	1.00 (0.99,1.01)	0.371	0.98 (0.97,1.00)	0.075
Body Pain	81.0 (22.5)	74.3 (22.6)	0.98 (0.97,0.99)	0.039	0.97 (0.94,0.99)	0.041
General Health	57.5 (13.1)	52.9 (13.5)	0.97 (0.95,0.99)	0.016	0.96 (0.93,0.98)	0.032
Vitality	66.9 (16.5)	62.0 (17.3)	0.98 (0.96,0.99)	0.042	0.98 (0.95,1.01)	0.211
Social Functioning	78.0 (21.9)	73.2 (20.2)	0.99 (0.98.1.00)	0.128	1.02 (0.98,1.04)	0.312
Role Emotional	23.8 (41.8)	22.0 (39.4)	0.99 (0.98,1.00)	0.760	0.99 (0.97,1.02)	0.116
Mental Health	69.7 (14.0)	63.2 (15.0)	0.94 (0.90,0.97)	< 0.001	0.95 (0.91,0.98)	0.005
Physical Health	61.5 (8.6)	57.8 (9.1)	0.96 (0.93,0.98)	0.004	0.94 (0.90,0.98)	0.026
Total SF-36	60.1 (6.6)	56.1 (7.4)	0.92 (0.88,0.96)	< 0.001	0.94 (0.89,0.98)	0.024
Social dysfunction	17.1 (1.8)	16.7 (2.4)	0.93 (0.82,1.05)	0.247	0.71 (0.47,1.08)	0.110
Anxiety Depression	11.4 (1.3)	11.3 (1.6)	0.95 (0.78,1.15)	0.595	1.51 (0.83,2.40)	0.177
Loss of confidence	5.7 (0.67)	5.6 (0.84)	0.86 (0.60,1.25)	0.441	0.94 (0.30,2.93)	0.912
Total GHQ	34.2 (3.7)	33.7 (4.7)	0.97 (0.90,1.04)	0.364	1.00 (0.92,1.09)	0.951

^a^Correctly classified = 83.48% and ROC curve = 0.74 for multivariable logistic regression model

## Discussion

COVID-19 appears to be contained by restrictive measures implemented in some nations throughout the world [34]. These measures, on the other hand, have interrupted people's regular jobs and activities, which might have serious consequences for their health and well-being [35].

This study aimed to investigate the relationship between the perception of risk of Covid-19 and the quality of life of the elderly population. The results of our study showed that 83% of elderly population was engaged in danger control responses and 17% in fear control responses indicating that a fifth of our elderly participants had negative discriminating values. Since, most of elderly populations have adequately higher perceptions of efficacy to counteract their threat perceptions to continue motivating these older people to engage in COVID-19 self-protective behaviors, it is necessary to emphasis on the susceptibility of target population and the severity of the COVID-19 threats [36]. In the similar study [23], older participants had higher self-efficacy sores than other group of populations for COVID-19 prevention. Self-efficacy is part of a person’s attitudes, abilities, and cognitive skills reducing stress and tension [37]. This study was conducted during fourth wave (between February and April 2021) of COVID-19 pandemic in Iran while the country battles high infection rates with tighter restrictions [38]. Despite the fact that older individuals experience greater negative outcomes during quarantines and social isolation for COVID-19 [39], older people exhibited increased self-efficacy [40]. This finding may be interpreted by adoption of social isolation and loneliness of older individuals prior COVI-19 pandemic. Additional explanation return to personal characteristics of older individuals for better resilience and getting appropriate coping strategy in the case of crisis and stress full events [41].

According to the results of this study, strong associations of fear control were found with being female, being illiterate, and having a good economic status and also body pain, general health, physical health and total quality of life were significantly associated with danger control. Findings of the study suggested that illiterate elderly people in the study setting were seven time more likely to engage in COVID-19 fear control. Consistent with our results, a study from Iran reported that the highest COVID-19 preventive behaviors related to people with higher education level [42]. Economic status plays an important role in adopting of preventive behaviors. Many COVID-19 behaviors such as wearing masks, eating healthy foods, regularly washing hands and disinfection of surfaces need to spend money [43]. Older individuals with low economic status and illiterate may receive inadequate care and family support. Our finding further highlights the importance of support services and facilities for mentioned older people.

The findings of this study showed that the variables of efficacy, denial, defend, resistance and, susceptibility were significantly different in the two age groups as among older population those who were 60–70 years had higher scores than 71 years and more. Although during the Covid-19 crisis, it was reported that people with chronic health problems were vulnerable groups with a poorer quality of life [44], in our study diabetes, or hypertention or CAD factros were not associted with fear control.

The results of our study consistent with previous research conducted during the pandemic investigated more characteristics of older individuals who were susceptible and at significant risk for their health [45, 46]. Because risk perception is inextricably connected to both psychological well-being and quarantine procedure observance [47, 48], It's important to look at risk perception and the elements that have previously been identified in the literature in the older population.

The fear control of COVID-19 is not directly related to physical and social functioning due to several factors. Fear control is an emotional response to Covid-19 threats, and it can vary significantly among individuals regardless of their physical or social capabilities. Someone who is physically fit and socially active may still experience fear and anxiety about Covid-19, while others who have limitations in physical or social functioning may not necessarily exhibit higher levels of fear [49, 50]. Additionally, fear control involves various psychological and cognitive processes that are influenced by factors such as personal beliefs, information exposure, and past experiences. These factors can shape an individual's perception of risk and their ability to manage fear, irrespective of their physical or social abilities. It is important to address fear and anxiety related to COVID-19 through targeted interventions that consider the diverse psychological and emotional aspects of individuals, rather than solely focusing on their physical or social functioning [51, 52].

As a result, prior experience dealing with various epidemics, such as influenza and infectious illnesses like AIDS and hepatitis, may help physicians with more past job experience be more effective [53]. People who feel they are capable of doing the appropriate reaction, for example, “It is simple for me to utilize disinfectants” or “It is simple for me to obtain masks and disinfectants,” have a high sense of self-efficacy, they also feel that following health advice is beneficial in avoiding Coronavirus” or “staying at home” (recommended response) works in averting the coronavirus threat, they have high perceived response efficacy. It appears that people's intense perceptions of the threat and efficacy of Coronavirus encourage them to take steps to mitigate the risk [23]. Denial of sickness is an ineffective coping technique for dealing with the issues that come with a chronic illness, including the greater risk of refusing to recognize the need for lifestyle adjustments [54]. It is crucial to identify patients who have a high incidence of denial to predict poor treatment adherence [55] and to devise strategies that make it easier for them to follow medical advice [29, 56]. Denial may have a helpful impact on decreasing anxiety in some circumstances [57]. High denial indices allowed patients with end-stage renal illness to sleep better, had fewer personality changes, and have fewer interpersonal issues [58]. Furthermore, Dimsdale [59] found that patients in intensive care units who scored higher on the denial scale had a higher chance of surviving. Denial, on the other hand, allows for evasive kinds of non-adherence in illnesses like Type 2 DM, which can have serious implications [57]. This result is in line with other studies conducted during the epidemics that reported a decrease in risk perception in the oldest-old [60, 61].

The COVID-19 pandemic is a huge public-health danger, with elderly people in particular at risk of serious health repercussions [62, 63]. The results of our studies showed that physical function is significantly different between the two groups and more in the age group of 60–70 years. The Role-physical variable is significantly different between the two groups (*p* = 0.001), and more in the age group of 71 years and older. This result is in line with the study by Won that analyzed the correlation between physical function and HRQOL and discovered that improved physical function enhances the quality of life [64].

### Strengths and limitations

One of the main strengths of this study is its ability to capture a snapshot of the predictors of fear control related to COVID-19 among older population at a given point in time of Covid-19 pandemic, providing valuable insights into fear control of Covid-19. Additionally, this study explore associations between socio-demographic factors, aiding in identifying potential risk or protective factors for Covid-19 among aging individuals during the Covid-19 pandemic. However, there are limitations to consider. Since data is collected from aging people at a single time point of Covid-19 pandemic, this study may be subject to recall bias or inaccuracies in self-reporting, as participants may struggle to recall or report accurate information about their experiences during the pandemic. Another limitation is the potential for selection bias, as participants may not be representative of the entire aging population, potentially affecting the generalizability of the findings.

## Conclusion

Strong associations of fear control were found with being female, being illiterate, and having a good economic status and also body pain, general health, physical health and total quality of life were significantly associated with danger control. The results of our study showed that 83% of elderly population was engaged in danger control responses and 17% in fear control responses indicating that a fifth of our elderly participants had negative discriminating values. Since, most of elderly populations have adequately higher perceptions of efficacy to counteract their threat perceptions to continue motivating these older people to engage in COVID-19 self-protective behaviors, it is necessary to emphasis on the susceptibility of target population and the severity of the COVID-19 threats. This study shows that perceived risk perception of Covid-19 varies in different age groups and is lower in oldest-old adults. Therefore, paying attention to this group of people is necessary for prevention and care measures.

## Data Availability

The data collection tools and datasets generated and/or analyzed during the current study are available from the corresponding author on reasonable request.

## Abbreviations

EPPM: Expanded parallel process model
HQOL: Health related quality of life
WHO: World health organization
ROC: Receiver operating characteristic
ANOVA: Analysis of variance
CAD: Cardiovascular disease
